# Age-dependent functional and transcriptional differences in intestinal mesenchymal stromal cells during early postnatal development in piglets

**DOI:** 10.3389/fcell.2026.1847165

**Published:** 2026-08-04

**Authors:** Hammed Ayansola, Younggeon Jin

**Affiliations:** Department of Animal and Avian Sciences, University of Maryland, College Park, MD, United States

**Keywords:** intestinal mesenchymal stromal cells, organoid coculture system, bulk RNA sequencing, calcium channel signaling, postnatal intestinal development, preweaned piglets

## Abstract

**Introduction:**

Intestinal mesenchymal stromal cells (iMSCs) regulate postnatal epithelial development, yet their temporal dynamics remain poorly defined in early postnatal piglets. Here, we investigated functional and transcriptional changes in jejunal iMSCs isolated from 0-, 7-, and 21-Day-old piglets.

**Methods and Results:**

In an enteroid-iMSC coculture system, Day 0 iMSCs supported predominantly cystic enteroids with the highest surface area and proliferation index, whereas Day 7 iMSCs increased multi-budded enteroid morphology with intermediate proliferation, showing age-dependent differences in iMSC-mediated epithelial growth modulation. Relative real-time qPCR revealed that these functional differences aligned with niche-associated expression patterns in iMSCs. Specifically, RSPO3 level was highest at Day 0, BMP4 and PDGFRα expression peaked at Day 7, and WNT and BMP antagonists (SFRP1 and GREM1, respectively) increased in Day 21 iMSC samples. Bulk RNA sequencing further revealed shifts in iMSC transcriptomes across developmental stages, with Day 7 iMSCs exhibiting a transitional transcriptional profile enriched for genes associated with cytoskeletal reorganization and cell fate regulation compared with Day 0 iMSCs. Notably, pathway enrichment and gene set analyses identified multiple calcium channel genes and related pathways in Day 7 iMSCs compared with Day 0 iMSCs. Short-term pharmacological inhibition of calcium channels in Day 7 iMSCs was associated with rapid changes in niche factor levels, including decreased BMP4 and increased RSPO3 and WNT expressions.

**Conclusion:**

Together, these findings suggest age-related functional and transcriptional differences in iMSCs across early postnatal intestinal development. Our results highlight that calcium channel-related processes may be associated with changes observed in the Day 7 iMSC profile, providing a framework for future studies on iMSC-epithelial interactions during early intestinal development in piglets.

## 1 Introduction

Piglets are born with an immature gut that must rapidly transform from a highly proliferative state to a more differentiated and functionally mature mucosa supporting nutrient absorption and mucosal defense (Modina et al., 2021; Duarte and Kim, 2022). This early developmental window is marked by striking morphological growth, such that their intestinal tissues double in weight and increase by 30% in length within the first 3 days after birth (Westrom et al., 2020; Modina et al., 2021). The intestinal epithelium in early postnatal (Day 0–7) piglets contains high proportions of undifferentiated SOX9^+^ and PCNA^+^ cells (Verdile et al., 2019), whereas later postnatal epithelial cells include abundant differentiated enterocytes marked by villin and FABP2 expression, alongside mucus-secreting goblet cells (MUC2^+^) and enteroendocrine cells (CHGA^+^) (Gonzalez et al., 2013; Meng et al., 2021; Yin et al., 2022). These postnatal structural and physiologic changes reflect coordinated maturation of the epithelium in response to nutritional, microbial, immune, and stromal signals (Meng et al., 2021; Duarte and Kim, 2022; Luo et al., 2022; Yin et al., 2022). Within this epithelial microenvironment, the intestinal mesenchymal stromal cells (iMSCs) contribute to tissue remodeling and epithelial renewal by providing essential niche factors (Ayansola et al., 2024). Although porcine studies have characterized intestinal epithelial crypt-villi cellular identities (Verdile et al., 2019; Meng et al., 2021; Yin et al., 2022), immune modulation (Tang et al., 2022), and microbial populations (Luo et al., 2022) during postnatal development, the contributions of iMSCs in piglets remain unknown, despite their recently appreciated roles in mouse intestinal maturation (Jacob et al., 2022).

iMSCs constitute a heterogeneous population of non-epithelial, non-endothelial, and non-hematopoietic cells, including fibroblasts, myofibroblasts, and smooth muscle cells (Roulis and Flavell, 2016; Soundararajan and Kannan, 2018; Ayansola et al., 2024). Historically viewed as structural supporters of the lamina propria (Brugger and Basler, 2023), iMSCs are now recognized as critical regulators of postnatal gut development (Greicius et al., 2018; McCarthy et al., 2020; Jacob et al., 2022). In mice, distinct iMSC subsets secrete spatially organized gradients of morphogens such as *RSPO3 (R-spondin 3)*, *WNT2B*, *GREM1 (Gremlin 1)*, and *BMP2/4* that sustain intestinal stem cell (ISC) proliferation within the crypt while promoting differentiation along the villus (Stzepourginski et al., 2017; Jacob et al., 2022; Liu et al., 2022; Kraiczy et al., 2023; McCarthy et al., 2023). In addition, the emergence of Ltβr^+^PDGFRα^hi^ iMSCs after birth further contributes to crypt confinement of stemness, epithelial specialization, and immune cell recruitment (Jacob et al., 2022), highlighting the heterogeneity of the stromal niche in shaping postnatal gut maturation. In contrast to mice, the temporal changes in iMSCs during early postnatal development in piglets have not been characterized despite growing interest in porcine models for neonatal intestinal development (Pohl et al., 2015; Ma et al., 2022).

Early postnatal development, therefore, represents the critical window during which the intestine transitions from a relatively immature state at birth to a more functionally specialized organ (Skrzypek et al., 2007; Modina et al., 2021). Key developmental differences between murine and porcine, including prolonged preweaning growth, delayed villus maturation, and distinct nutrient- and microbial-driven signaling before weaning in piglets, necessitate species-specific investigation (Gonzalez et al., 2015; Ziegler et al., 2016; Burrin et al., 2020; Lim et al., 2020; Yang et al., 2025). Whether porcine iMSCs undergo temporal and molecular changes as described in mice remains unknown in piglets (Stzepourginski et al., 2017; Greicius et al., 2018; McCarthy et al., 2020; Jacob et al., 2022; Kraiczy et al., 2023; Manieri et al., 2023; McCarthy et al., 2023; Paerregaard et al., 2023; Ayansola et al., 2024). Because the jejunum is a major site for nutrient absorption and is highly susceptible to early life challenges such as weaning stress, it provides an ideal region for investigating stromal dynamics during postnatal development in piglets (Modina et al., 2021; Ma et al., 2022).

Using enteroid-iMSC coculture assays, qPCR, and bulk RNA sequencing, we characterized age-associated differences in the functional and transcriptional profiles of jejunal iMSCs isolated from 0, 7, and 21-Day-old piglet jejunum. We identified age-related shifts in niche factor gene expression, enteroid growth support capacity, and transcriptome profile, with Day 7 iMSCs exhibiting a transcriptional profile enriched for calcium channel-associated gene signatures. We further examined the effects of pharmacological calcium channel inhibition on niche factor expression in Day 7 iMSCs, providing a framework for future investigations of stromal-epithelial interaction during early intestinal development in piglets.

## 2 Materials and methods

### 2.1 Animal ethics

The animal management procedure R-JUN-22-30 was reviewed and approved by the University of Maryland’s Institutional Animal Care and Use Committee (IACUC). All experimental procedures involving animals were performed in accordance with relevant institutional guidelines and regulations for the care and use of laboratory animals, including the NIH Guide for the Care and Use of Laboratory Animals. This study is reported in accordance with the ARRIVE guidelines 2.0 for reporting animal research.

### 2.2 Experimental design

The study was conducted at the pig facility of the University of Maryland, College Park. For this research, we purchased a commercially raised, 90-Day-old pregnant Yorkshire (*S. scrofa*) sow, fed and housed her until she farrowed on gestation Day 117. After farrowing, the piglets were weighed and tagged for proper identification and management. Piglets were cleaned and allowed to suckle until the time points for tissue sample collection. A total of 12 mixed sex piglets were randomly divided into three age groups: Day 0, Day 7, and Day 21. We selected 4 piglets per age as biological replicates for each group based on their average birth weight. The piglets in each age group were euthanized with captive bolts, and the proximal jejunum tissue samples were collected.

### 2.3 Tissue sample collection

On the day of sample collections for each age group, we excised the proximal jejunum segment, dissected it longitudinally, washed the luminal content with cold 1X PBS, and cut each segment into four parts. We immediately snap-froze one section in liquid nitrogen and stored each tube at −80 ^0^C. The remaining three parts were placed into 50 mL tubes containing 30 mL cold 1X PBS for further experimental procedures, including cell isolations and tissue immunofluorescent staining.

### 2.4 Intestinal epithelial and mesenchymal stromal cell isolation and *in vitro* cell culture

This study marks the first isolation of iMSCs from porcine tissue. Jejunal samples were collected from three age groups (Days 0, 7, and 21) with four biological replicates per group. Our recently published protocol describes a modification of an established adult mouse procedure to optimize the sequential isolation of intestinal epithelial and adherent mesenchymal stromal cells from the same jejunal segment (Koliaraki and Kollias, 2016; Ayansola and Jin, 2025). Briefly, the optimized procedure includes two sequential phases: epithelial layer dissociation and extracellular matrix (ECM) digestion. Tissues were washed in wash buffer (1% anti-anti in 1X PBS) until the solution was clear, then cut into ∼10 mm sections to increase surface area before dissociation. We incubated the minced tissues in 30 mL of prewarmed epithelial dissociation buffer (1x PBS, 5 mM EDTA, 1% anti-anti, and 1 mM DTT) for ∼30 min at 37 °C, with intermittent shaking. After incubation, the dissociation solution was aspirated and replaced with wash buffer, and then the suspended crypt segments were collected through a 100 μm cell strainer. The subepithelial remnants were retained on ice for subsequent iMSC isolation, while the isolated crypts were centrifuged and resuspended in pig enteroid culture media containing 50% L-WRN conditioned media (ATCC, CRL-3276), Advanced DMEM/F12 (Gibco, 12634010), 1% Anti-Anti (Gibco, 15240096), 1X B-27 (Gibco, 12587010), 50 ng/mL EGF (PeproTech, AF-315-09), 10 nM Gastrin (Sigma, G9020-0.5 MG), 1X GlutaMaX (Gibco, 35050061), 10 nM HEPES (Gibco, 15630080), 1.25 mM N-acetyl cysteine (MP Biomedicals, 0219460305), 10 mM Nicotinamide (Sigma-Aldrich, N0636-100G), 0.1 µM PGE2 (Cayman chemical, 14010), 3 µM SB202190 (AdipoGen, AG-CR1-0028-M025), and 10 µM Y27632 (Apexbio, A300810). The resuspended crypts were then seeded on 1 mg/mL collagen-coated plates and cultured with media changes every other day to expand as 2D epithelial enteroid monolayers, as presented in our published protocol (Ayansola and Jin, 2025).

Following crypt isolation, the subepithelial tissue remnants were washed twice to remove residual EDTA before incubation in 25 mL of ECM digestion buffer (1.1 mg/mL Collagenase IV and 0.1 mg/mL Dispase II in high-glucose DMEM) for 20 min at 37 ^o^C to release iMSCs from the ECM (Ayansola and Jin, 2025). The supernatant containing the released iMSCs was decanted through a 100 μm cell strainer, verified via microscopy, and centrifuged to pellet the cells. The cell pellets were then resuspended in iMSC media (10% FBS, 1% Anti-Anti, 10 μg/mL gentamicin in high-glucose DMEM), plated in T75 flasks, and verified to adhere within 24 h (Ayansola and Jin, 2025). The culture medium was changed every other day until day 6, when the cells reached 90% confluence and were ready for subsequent experimental use. These cultured iMSCs were defined as adherence-enriched intestinal mesenchymal stromal cells (iMSCs). Immunostaining confirmed the expression of broad mesenchymal markers, including PDGFRα, CD81, and Vimentin, consistent with our published isolation protocol (Supplementary Figure S1) (Powell et al., 2011; Roulis and Flavell, 2016; Ayansola and Jin, 2025).

### 2.5 Intestinal epithelial-mesenchymal stromal cells 3D direct coculture assays

To investigate the age-dependent effects of postnatal iMSCs on enteroid growth behavior, 7-Day-old jejunum-derived enteroids were directly co-embedded with 5000 iMSCs isolated from the three age groups: 0, 7, and 21-Day-old jejunum. The enteroid-only group was also prepared similarly without iMSCs and served as controls.

For enteroid fragment preparation, established 2D enteroid monolayers were passaged using the stepwise collagenase-EDTA epithelial monolayer passaging procedure. Briefly, we removed the expansion medium from the well without disrupting the underlying collagen scaffold. The collagen scaffold with the attached enteroid monolayers was scraped and transferred to a 15 mL conical tube, followed by the addition of 100 µL collagenase IV (Gibco, 17104019) to digest the collagen matrix at 37 °C for 10 min in a bead bath. The digestion was quenched with 1 mL of 10% FBS/PBS solution, and the suspended epithelial fragments were centrifuged at 600 × g for 1 min at room temperature. The fragment pellet was washed twice with 10% FBS/PBS solution, then resuspended in 1 mL PBS containing 1 µL of 0.5 mM EDTA and 1 µL of 10 mM Y27632 and incubated at 37 °C for 5 min. After incubation, the fragments were further dissociated by pipetting up and down with a p200 pipette tip, centrifuged at 600 × g for 1 min, and resuspended in the culture medium. The dissociated fragments were selectively aspirated using a p10 pipette tip to recover epithelial fragments of approximately 35 µm while excluding larger fragments, ensuring uniform fragment sizes for 3D embedding.

For iMSCs preparation, passage 2 iMSCs were washed with 1x PBS and dissociated using 3 mL of 0.05% trypsin/1 mM EDTA solution (Corning, 25-051-CI) for 5 min in the 37 °C incubator. Trypsin activity was quenched with twice the volume of iMSC media, and suspended cells were centrifuged at 600 × g for 2 min at room temperature. The supernatant was aspirated, the cell pellet was resuspended in iMSC media, and viable cells were counted using a hemocytometer with trypan blue exclusion.

**FIGURE 1:**
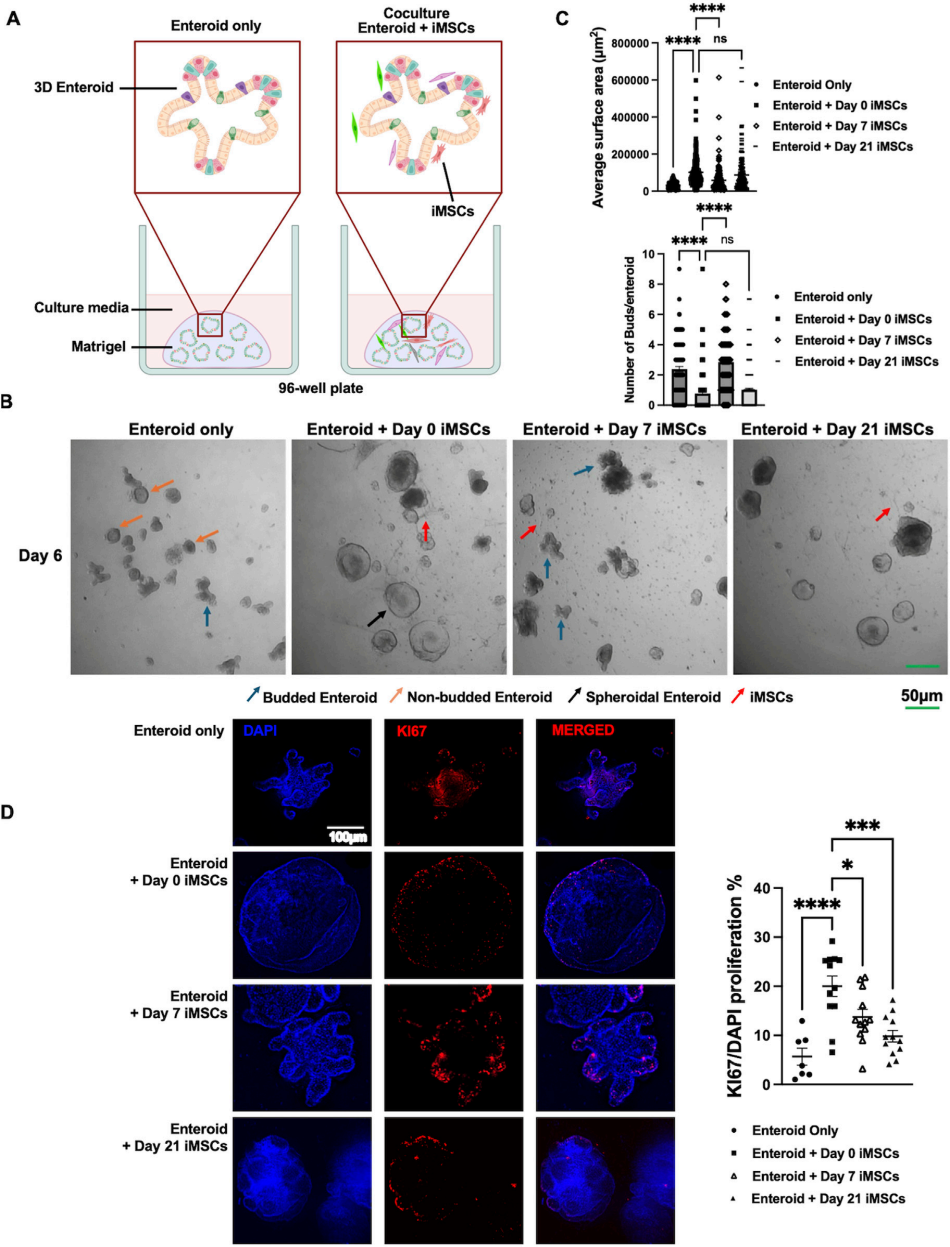
Age-dependent effects of postnatal iMSCs on enteroid growth and proliferation patterns. **(A)** Schematic illustration of the 3D direct coculture experimental design. Day 7 jejunal enteroid fragments were co-embedded with iMSCs isolated from Days 0, 7, and 21 piglet jejunum in Matrigel droplets and evaluated at Day 6 post-seeding. Enteroid-only controls were prepared identically without iMSCs. **(B)** Representative brightfield images of piglet jejunal-derived enteroids cultured alone or co-cultured with iMSCs from the different age groups. Orange arrows, non-budded enteroids; blue arrows, budded enteroids; black arrows, spheroidal enteroids; and red arrows, iMSCs. Scale bar = 50 µm. **(C)** Quantification of average enteroid surface area (left) and number of buds per enteroid (right) at day 6 post-seeding. Individual data points represent individual enteroids; bars show mean ± SEM. Enteroid fragments were derived from a single Day 7 piglet and were used as technical replicates for the enteroid (a minimum of 100 enteroids were scored per group). Cocultured iMSCs were isolated from n = 4 individual piglets per age group (biological replicates). One-way statistical test followed by Sidak multiple comparisons test. ***p < 0.0002, ****p < 0.0001, and ns represent no statistical differences. **(D)** Representative KI67/DAPI immunofluorescence images of enteroids at Day 6, showing DAPI nuclear counterstain (blue), KI67 proliferation marker (red) with merged overlay and the quantification of the KI67/DAPI proliferation index % of enteroids per imaging field for each group; bars show mean ± SEM. Each enteroid fragments across all groups were derived from the same single Day 7 piglet; each dot represents one imaging field. Statistical analysis was performed using a one-way ANOVA followed by Dunnett’s multiple comparisons test. ****p < 0.0001 (Enteroid + Day 0 iMSCs vs. enteroid only); *p < 0.05 (Enteroid + Day 0 iMSCs vs. Enteroid + Day 7 iMSCs); ***p < 0.001 (Enteroid + Day 0 iMSCs vs. Enteroid + Day 21 iMSCs).

Subsequently, we proceeded to the 3D coculture experiments by directly co-embedding enteroid fragments with iMSCs in ice-cold Matrigel (Corning, 356231). Each 10 µL Matrigel contained 15 enteroid fragments and 5000 iMSCs per well. Enteroid fragments derived from a single Day 7 piglet jejunum served as consistent replicates across all coculture groups, representing the technical replicates for the enteroid used in the morphometric and proliferation evaluations. The biological variable in the co-culture assays was the iMSCs, meaning that iMSCs were isolated from four individual piglets per age group (Days 0, 7, and 21), each representing an independent biological replicate. Using a p10 pipette tip, we dispensed the 10 µL embedded Matrigel droplets into individual wells of pre-warmed 96-well plates and allowed them to polymerize at 37 °C for 15 min (Figure 1A). After polymerization, enteroid culture medium, as described in Section 2.4, was added to all wells and maintained throughout the experiments. Enteroid surface area and number of buds per enteroid were evaluated at Day 6 post-seeding across all wells, with a minimum of 100 enteroids scored per group to compare the age-dependent effects of postnatal iMSCs on the enteroid growth behavior. For morphometric evaluation, enteroids were classified based on brightfield microscopy into three types: cystic (hollow, spherical, smooth epithelial ring surface), multibudded (two or more outward epithelial protrusions, consistent with the crypt-like domain formation), and non-hollow spheroidal shape with less defined lumen.

### 2.6 Total RNA isolation

We used a total of 4 biological replicates for each age group (Days 0, 7, and 21) for RNA isolation from the cultured iMSCs from passage 2, described in the *in vitro* cell culture procedure section. Following the Trizol reagent guide (Invitrogen, 15596026), we isolated total RNA from the cultured iMSCs of piglets’ jejunum and checked the concentration and purity levels using a Nanodrop 2000 spectrophotometer. Next, 1500 ng of total RNA was reverse transcribed into cDNA template for the quantitative real-time PCR assay (Quant Studio 5, Applied Biosystems) using Biorad iTaq Sybr® Green Supermix (1725122) to check the amplification plots. Target gene expression was normalized to the housekeeping gene *GAPDH* and presented on the graph as relative fold changes using the comparative 2^−Δ^ ^ΔCT^. Primer sequences were provided in Supplementary Table S1.

### 2.7 Immunofluorescence

For the immunofluorescence (IF) procedure, we fixed the rinsed jejunum tissues from all age groups (four biological replicates/group) in 4% paraformaldehyde for 24 h at 4 °C. The fixed tissues were transferred into a 30% sucrose solution and incubated for another 24 h at 4 °C. Next, we embedded the tissues in Tissue-Tek Optimal Cutting Temperature (OCT) using an OCT block to gradually freeze them in isopentane cooled with a liquid nitrogen solution. Following the established IF protocol, we sectioned the OCT-embedded tissues at 10 µm using a cryostat and mounted the sections on coated slides. Sectioned slides were washed three times with 1X PBS, heated in a 95 °C 1x citrate buffer solution (Biologend, 420902) for 20 min to expose the antigen surface, permeabilized with 0.3% Triton X-100/PBS (PBST), rinsed twice with PBST, and the permeabilized sectioned tissues were blocked with 5% BSA/PBST for 1 h. Primary antibodies diluted with Dako diluent were applied to the tissues and incubated overnight at 4 °C. We counterstained the primary antibodies with secondary antibodies and DAPI diluted in 1% BSA/PBST for 1 h at room temperature. Finally, the stained slides were rinsed with PBST and mounted with DAKO mounting solution. Immunostaining of the cultured iMSCs followed a similar procedure after cell fixation with 4% PFA, consistent with our published protocol (Ayansola and Jin, 2025). Antibody concentrations and reagents are listed in Supplementary Table S1. Immunostaining images were captured by immunofluorescent microscopy and processed with Fiji (ImageJ). We used the ImageJ mean fluorescence intensity function to further normalize and quantify the relative PDGFRα staining intensity.

### 2.8 Library preparation and RNA sequencing

RNA samples from the cultured iMSCs used for the library preparation had a 260:280 absorbance ratio of 2.0 and an RIN of 10. Four RNA samples for each age group, making a total of 12 RNA samples, were submitted for bulk RNA sequencing library preparation done by Innomics Inc. Using the DNBSEQ platform and paired-end 150 (PE 150) for the read length (RNA sequencing service number F24A480000179). Briefly, the process followed mRNA enrichment and purification, fragmentation and reverse transcription, adaptor ligation, and PCR amplification to prepare the cDNA library. The QC and differential expression analyses were performed using *DESeq2 (v1.4.5)*. The genome annotation was referenced to Sus_scrofa_9823 (NCBI.GCF_000003025.6_Sscrofa11.1v2201) with an average yield of 6.87 GB per sample. The average alignment ratio of the sample to the reference genome was 97.10%, while the average alignment of the gene set was 74.19% using HISAT and Bowtie2 as described in the Innomics sequencing service report. The data was further subjected to differential gene expression (DEGs), gene ontology, Kyoto Encyclopedia of Genes and Genomes (KEGG), and GSEA enrichment analyses using Dr. Tom’s online platform (https://biosys.innomics.com/#/report/login).

### 2.9 RNA sequencing and data analysis

Expression quantification was performed using Bowtie2 to map clean reads to the reference gene sequence and RSEM to calculate gene expression levels for each sample (Li and Dewey, 2011; Langmead and Salzberg, 2012). Finally, the DEGs reported in this study met the adjusted P-value (q-value ≤0.05) and a fold change ≥1 using DESeq2, except where stated (Love et al., 2014). The enrichment threshold of q-value ≤0.05 was met for the GO terms, KEGG pathway, and GSEA reports. The enrichment data were downloaded from Innomics Dr. Tom’s online platforms and are reported in the Supplementary Tables for the annotated gene sets, DEGs, GO, KEGGs, and GSEA. Principal Component Analysis (PCA) was performed to observe the clustering pattern among the 12 iMSC samples (n = 4 biological replicates per age group). For the PCA visualization, TPM expression values for all 16,492 annotated genes were standardized across samples (mean-centered and divided by the gene-wise standard deviation) before analysis. Hierarchical clustering was generated on Dr. Tom (https://biosys.innomics.com/#/report/login), and the visualization of selected genes for the expanded heatmap was done using ClustVis (https://biit.cs.ut.ee/clustvis), a web-based tool for clustering and visualization of multivariate data (Metsalu and Vilo, 2015). The row z-scores were calculated from log2 (TPM+1) values.

For cross-species contextual analyses, publicly available single-cell RNA sequencing data from the mouse postnatal intestinal mesenchyme (GSE184158; McCarthy et al., 2023, Developmental Cell) (McCarthy et al., 2023) were interrogated to examine the expression patterns of the three pig Day 7-upregulated calcium channel genes (TRPA1, CACNA1I, P2RX7) across five postnatal developmental stages (P1, P4, P5, P9, P14) and six intestinal mesenchymal cell types. Spearman correlation coefficients between calcium channel gene expression and ISC niche factor expression (Wnt2b, Rspo3, Bmp4, Bmp2, Grem1, Pdgfra, Foxl1) were computed across all cell types at mean and P5 stage-specific resolution (rho = 0.17–0.30, padj <0.001). Statistical significance was assessed after Benjamini-Hochberg false discovery rate correction. These analyses were performed to provide cross-species contextual data for the calcium channel findings observed in piglet iMSCs and are presented as Supplementary Figures S3-S5.

### 2.10 Statistical analysis

All enteroid morphometric and qPCR data quantification were statistically analyzed using GraphPad Prism 10 and are reported as mean ± SEM. Group differences were further tested with a one-way ANOVA and Sidak’s or Brown-Forsythe for multiple comparisons, as appropriate.

## 3 Results

### 3.1 Age-related iMSCs differentially alter enteroid growth morphology

To determine whether postnatal stromal age affects epithelial growth behavior in piglets, Day 7 jejunal enteroids were grown alone or cocultured with iMSCs from different postnatal ages (0, 7, and 21-Day-old), all maintained in the complete enteroid medium (Figure 1A). Enteroids cultured without iMSCs in this condition formed predominantly multi-budded structures but exhibited the smallest surface area, consistent with baseline epithelial growth under exogenous niche factor conditions (Figures 1B,C). In contrast, functional coculture assays revealed age-dependent effects of postnatal iMSCs on epithelial enteroid morphology and growth patterns, which generally produced larger enteroids than those in enteroid-only cultures, reflecting additional stromal support (Figure 1B). Day 0 iMSCs supported the formation of large cystic enteroids, whereas Day 7 iMSCs increased the proportion of multi-budded enteroids (Figures 1B,C). We observed that iMSCs from Day 21 generated mixed enteroid morphologies, including both cystic and budded structures. Notably, morphometric quantifications of the enteroid surface area and number of buddings confirmed these significant age-dependent differences of piglet iMSCs on enteroid growth (Figure 1C). To validate the morphological observations, Ki67/DAPI immunofluorescence confirmed that enteroid cocultured with Day 0 iMSCs exhibited the highest enteroid proliferation percentage compared to the enteroid-only control and those cocultured with Day 7 and Day 21 iMSCs (p < 0.001) (Figure 1D). Enteroid cocultured with Day 7 iMSCs showed an intermediate proliferation rate. Together, these findings are consistent with age-dependent functional differences in the capacity of postnatal iMSCs to support enteroid proliferation and epithelial growth behavior.

### 3.2 Age-dependent shift in iMSC niche-associated gene expression

**FIGURE 2:**
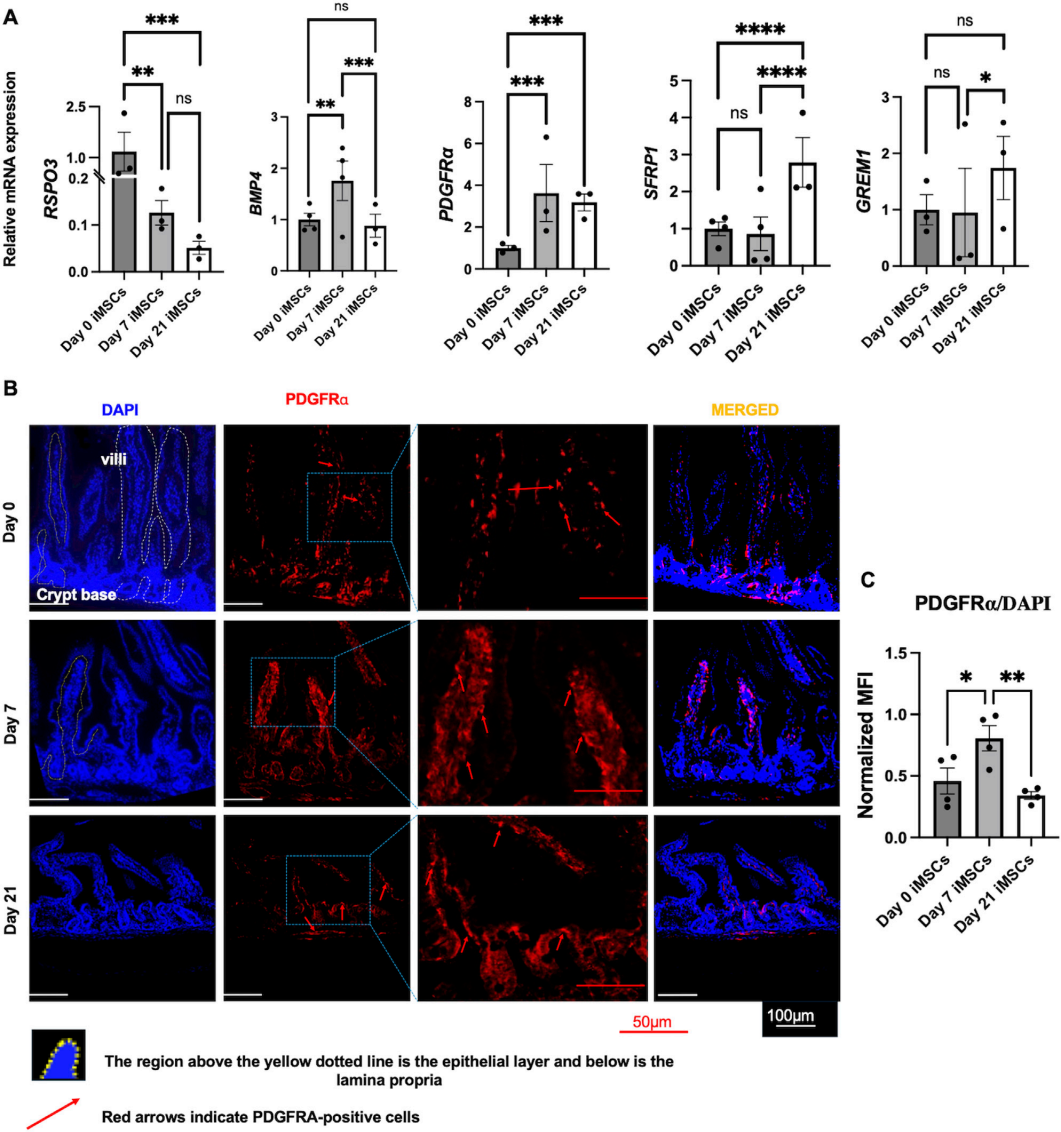
Temporal niche factor gene expression in postnatal piglet jejunal iMSCs. **(A)** Relative mRNA expression of *RSPO3*, *BMP4*, *PDGFRα*, *SFRP1*, and *GREM1* was quantified by qPCR and normalized to *GAPDH* using the ΔΔCt method. Bars show the mean expression ±SEM of n = 4 individual biological replicates per age group. Statistical comparisons were performed using one-way ANOVA with the Sidak multiple-comparison test. *p < 0.05, **p < 0.0015, ***p < 0.0007, ****p < 0.0001, and ns represent no statistical differences. Additional selected niche factor gene expressions are provided in the Supplementary Figure S2 file. **(B)** Representative immunofluorescence images of jejunal iMSC from 0-, 7-, and 21-Day-old piglets stained for DAPI nuclear counterstain (blue) and PDGFRα (red). White line tracing showing crypt-villi orientation. The yellow line tracing separates the epithelial and subepithelial zones. Scale bar = 100 μm. n = 4 biological replicates per age group. **(C)** Normalized mean fluorescence intensity of PDGFRα relative to DAPI. One-way ANOVA test followed by Dunnett’s multiple comparisons test (*p < 0.03, **p < 0.008).

To determine whether the functional differences observed in the coculture assay are associated with changes in niche signaling, we next quantified the expression of selected key stromal-derived factors in iMSCs from Days 0, 7, and 21 piglets using relative real-time PCR (qPCR). *RSPO3* mRNA was significantly upregulated in Day 0 iMSCs, consistent with the previously reported role of *RSPO3* as a Wnt-potentiating niche factor that promotes intestinal stem cell maintenance and epithelial expansion during neonatal growth (Greicius et al., 2018). In contrast, *BMP4* expression was significantly increased in 7-Day-old iMSCs relative to Day 0 and Day 21 (Figure 2A), aligning with the multibudded morphologies observed in enteroids cocultured with Day 7 iMSCs (Figure 1B) (McCarthy et al., 2020; Kraiczy et al., 2023). To further examine niche-related genes during the early neonatal-Day 7 transition, we additionally quantified *BMP2* and several WNT ligands (*WNT2, WNT4,* and *WNT5A*) in Days 0 and 7 iMSCs (Supplementary Figure S2). *BMP2, WNT2,* and *WNT4* showed numerically higher but nonsignificant higher relative expression level in Day 7 than Day 0, but *WNT5A* was significantly higher in Day 0 than Day 7 (P < 0.05).

Given recent evidence that *PDGFRα* ^*hi*^ stromal populations are associated with elevated *Bmp* ligand expression (McCarthy et al., 2020; Jacob et al., 2022; Kraiczy et al., 2023; Manieri et al., 2023), we next quantified *PDGFRα* gene expression among the age groups. *PDGFRα* gene expression was significantly increased in Day 7 iMSCs relative to Day 0 and Day 21, coinciding with the time point with the highest *BMP4* expression (Figure 2A). Moreover, immunofluorescence analyses of jejunal tissue sections revealed the presence of the expanded PDGFRα^+^ stromal population within the lamina propria across all age groups, with an increased signal intensity observed at Day 7, as determined by mean fluorescent intensity analysis (Figures 2B,C).

Additional niche-associated ligands showed that by Day 21, expression of WNT and BMP antagonists, *SFRP1* and *GREM1*, respectively, was significantly increased relative to earlier ages (Figure 2A), consistent with an increasingly complex niche landscape in which iMSCs express both proliferative and differentiation-associated signals (Kim et al., 2020; Brugger and Basler, 2023; Kayama and Takeda, 2024). Together, these stromal ligand data reveal dynamic molecular changes associated with postnatal development in piglets, consistent with the age-related functional differences observed in the coculture assays.

### 3.3 Bulk RNA sequencing identifies age-related transcriptional changes in iMSCs across the preweaning period

**FIGURE 3:**
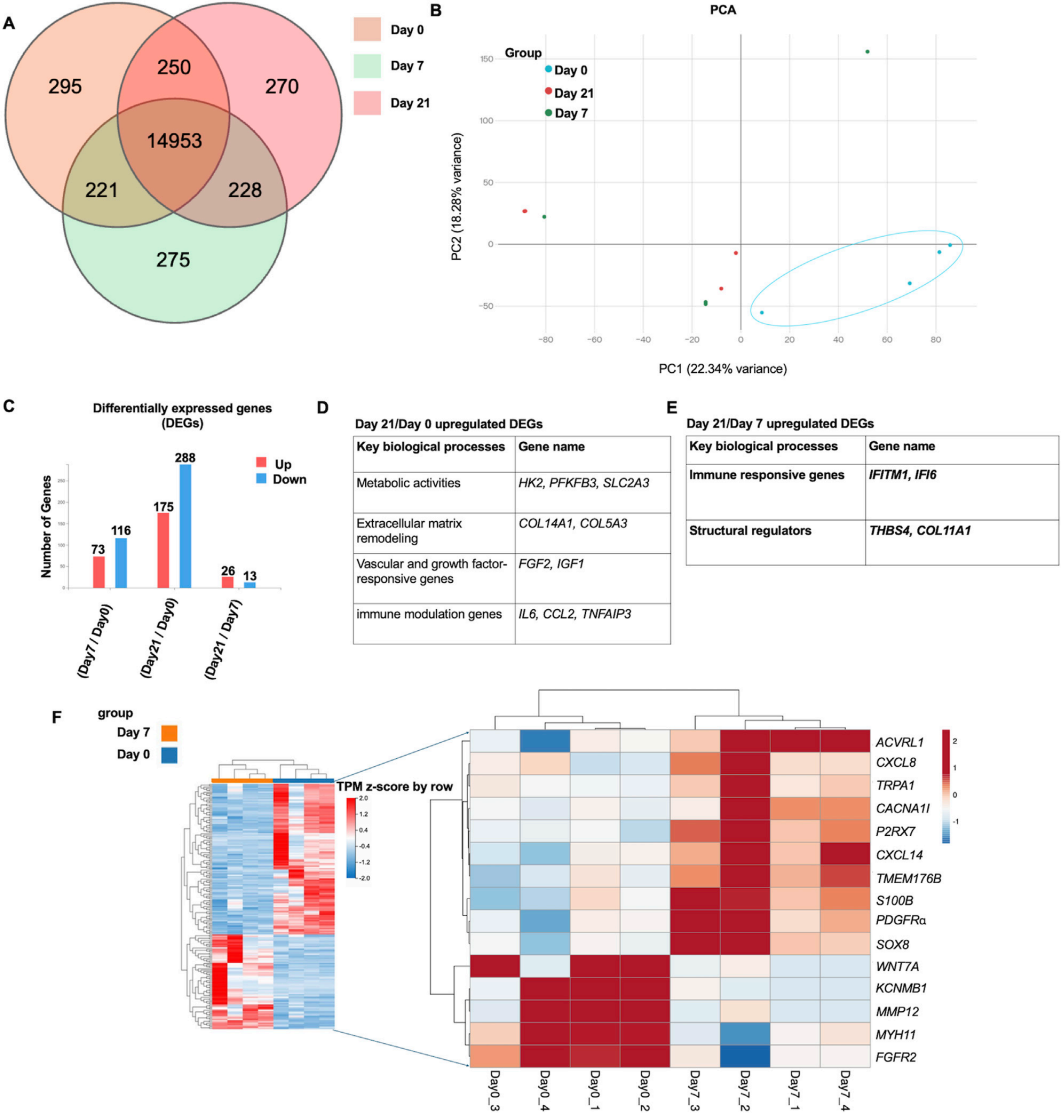
RNA-sequencing analyses reveal age-related transcriptional differences in iMSCs across the preweaning period. **(A)** Venn diagram showing overlap of expressed genes among Days 0, 7, and 21 iMSCs. Total annotated gene sets are provided in Supplementary Table S2. **(B)** PCA clustering of all annotated genes in preweaned jejunal iMSCs. Each dot represents one biological replicate per group. Colors indicate age groups (Day 0 = cyan, Day 7 = green, and Day 21 = red). The ellipse indicates the Day 0 replicate cluster. n = 4 biological replicates per age group. **(C)** Bar graph showing the number of statistically significant upregulated (red) and downregulated (blue) differentially expressed genes (DEGs; Log2FC ≥ 1, q ≤ 0.05) identified in pairwise comparisons (Day 7 vs. Day 0, Day 21 vs. Day 0, and Day 21 vs. Day 7). n = 4 biological replicates per group. Full DEGs are provided in Supplementary Table S3. Tables of selected upregulated DEGs and key biological processes from. **(D)** Day 21 vs. Day 0 comparison and **(E)** Day 21 vs. Day 7 comparison, respectively (Log2FC ≥ 1, q ≤ 0.05). **(F)** Hierarchical clustering heatmap of all 189 statistically significant DEGs from the Day 7 vs. Day 0 comparisons (Log2FC ≥ 1, q ≤ 0.05). Rows show individual genes (z-scored log2TPM+1 values), columns show individual biological replicates. Group color bars: orange = Day 7 iMSC samples and blue = Day 0 iMSC samples.

Bulk RNA sequencing was performed to characterize the broader transcriptomic differences in iMSC from 0, 7, and 21-Day-old piglets during the preweaning stages. A total of 16,492 expressed genes were annotated (Supplementary Table S2), of which 14953 were shared among all time points, whereas 295, 275, and 270 genes were uniquely expressed in 0, 7, and 21-Day-old iMSCs, respectively (Figure 3A). Using the annotated gene sets, PCA visualised the clustering patterns among the iMSC replicates across the three age groups (Figure 3B). The first two principal components (PC1 = 22.34% variance and PC2 = 18.28% variance) reveal that Day 0 iMSCs formed a distinct cluster along the positive PC1 axis, separated from the majority of Day 7 and Day 21 samples, reflecting a neonatal iMSC transcriptional profile distinct from later preweaning stages. Day 7 and Day 21 iMSC samples showed greater within-group variability and partially overlapped in the PC1/PC2 landscape, suggesting age-dependent transcriptional shifts across the preweaning period. The within-group dispersion among Day 7 and Day 21 iMSCs replicates is consistent with expected *in vivo* variability among individual animals, although 1 Day 7 replicate showed a notably distinct PC2 position relative to the other three (n = 4 per group).

We identified 691 statistically significant differentially expressed genes (DEGs) encoding mRNA among all the timepoints (log2FC ≥ 1, q-value ≤0.05 – adjusted p-value that accounts for the false discovery rate) (Figure 3C). The highest number of DEGs (463: 175 upregulated and 288 downregulated) was recorded between Days 21 and 0 (Day 21/Day 0), followed by 189 DEGs (73 upregulated and 116 downregulated) between Days 7 and 0 (Day 7/Day 0), and the lowest number of DEGs (39: 26 upregulated and 13 downregulated) were observed between Days 21 and 7 (Day 21/Day 7) (log2FC ≥ 1, q-value ≤0.05). Notably, nearly five times more DEGs distinguished Day 7 from Day 0 than distinguished Day 21 from Day 7, suggesting that a larger proportion of the transcriptional divergence occurs within the first postnatal week, consistent with Day 7 representing a transitional stage along the postnatal developmental trajectory.

Comparing Day 21 with Day 0 iMSCs revealed a transcriptional profile enriched for genes linked with metabolic activities (*HK2, PFKFB3, SLC2A3*), extracellular matrix (*COL14A1, COL5A3*), vascular and growth factor-responsive genes (*FGF2,* IGF1), and immune modulation gene sets (*IL6, CCL2, TNFAIP3*) (Figure 3D). Analyses of upregulated DEGs between Day 21 and Day 7 identified genes primarily linked to immune (e.g., *IFITM1, IFI6*) and structural regulators (e.g., *THBS4, COL11A1*), pointing toward an increasing stromal immune signal responsiveness at later preweaning stages (Figure 3E) (Peiris et al., 2007; Meng et al., 2021; Jacob et al., 2022; Paerregaard et al., 2023; Chalmers et al., 2025).

Hierarchical clustering of the 189 Day 7 vs. Day 0 DEGs grouped all Day 7 and Day 0 iMSC replicates by age (Figure 3F). Among the upregulated DEGs in Day 7 were genes encoding ion channels (including the calcium-permeable *TRPA1, CACNA1I,* and *P2RX7*), calcium homeostasis regulator (*TMEM176B*), mesenchymal marker (*PDGFRα*), chemokines (*CXCL8, CXCL14*), and distinct cell fate regulators associated with neuroglial specification (*SOX8, S100B*)*,* and vascular differentiation (*ACVRL1*). In contrast, the downregulated DEGs in Day 7 iMSCs included Wnt ligand (*WNT7A*), ECM-degrading proteinase (*MMP12*), and growth factor receptor (*FGFR2*) genes. Together, these findings point to an age-related transcriptional shift away from neonatal proliferative-associated genes by Day 7 during early postnatal intestinal development in piglets (Supplementary Table S3).

**FIGURE 4:**
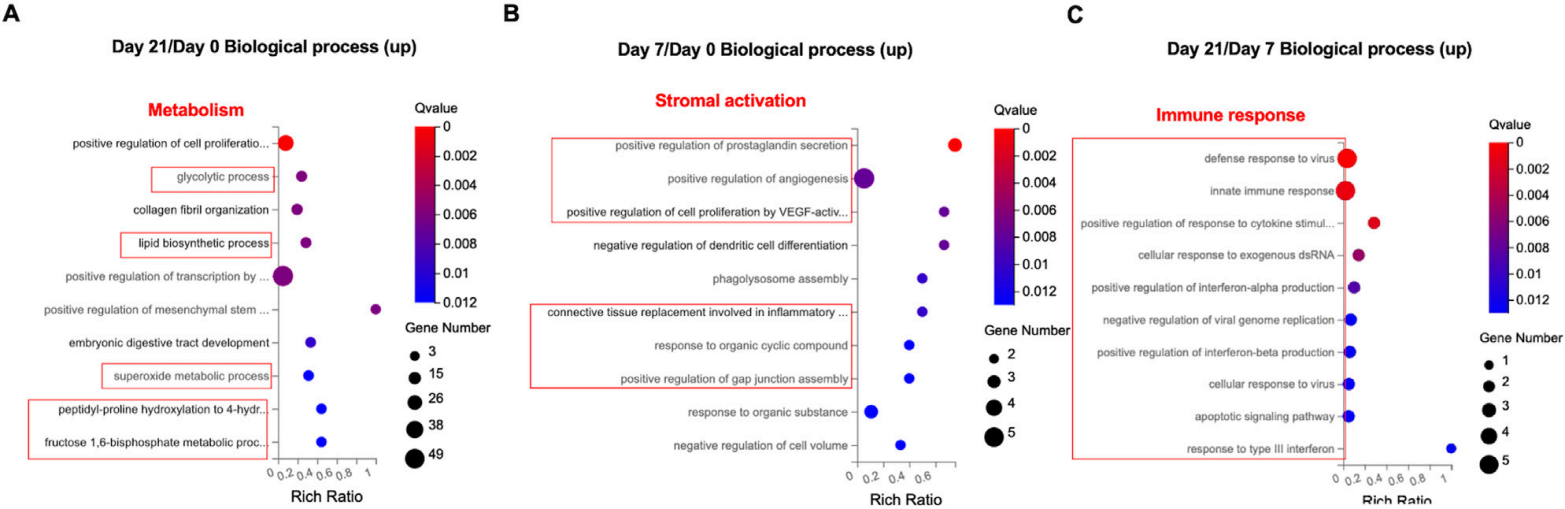
Gene ontology enrichment across iMSCs from different preweaning age groups. **(A–C)** Top significantly upregulated GO biological process terms (ranked by enrichment ratio, q ≤ 0.05) for Day 21 vs. Day 0 **(A)**, Day 7 vs. Day 0 **(B)**, and Day 21 vs. Day 7 **(C)** comparisons. Color intensity indicates the statistical significance level. Full GO terms are provided in Supplementary Table S4. N = 4 biological replicates per group.

Gene Ontology (GO) enrichment analyses corroborated these transcriptional patterns in postnatal iMSCs. For the Day 21 vs. Day 0 comparison (q-value ≤0.05; ranked by enrichment ratio), enriched biological processes shifted toward development (positive regulation of cell proliferation and digestive tract development), metabolism (glycolysis process and lipid biosynthesis), differentiation (positive regulation of mesenchymal stem cell differentiation), and tissue reorganization (collagen fibril organization) (Supplementary Table S4; Figure 4A). For the Day 7 and Day 0 comparisons, significantly enriched biological processes consist of early stromal activation pathways, including positive regulation of prostaglandin secretion, positive regulation of angiogenesis, chemotactic signaling, and calcium ion transport/import into the cytosol (Supplementary Table S4; Figure 4B). Biological processes enriched in the Day 21 vs. Day 7 upregulated gene sets were mainly related to regulation of immune activities, such as positive response to cytokine stimulus, defense response to virus, and apoptotic signaling regulations (Supplementary Table S4; Figure 4C). Collectively, these GO enrichment patterns are consistent with an early proliferation signal-responsive profile at pre-Day 7, followed by metabolic, ECM, differentiation, and immune response processes by Day 21.

### 3.4 KEGG and GSEA identify enriched calcium channel-associated pathways in day 7 iMSCs

**FIGURE 5:**
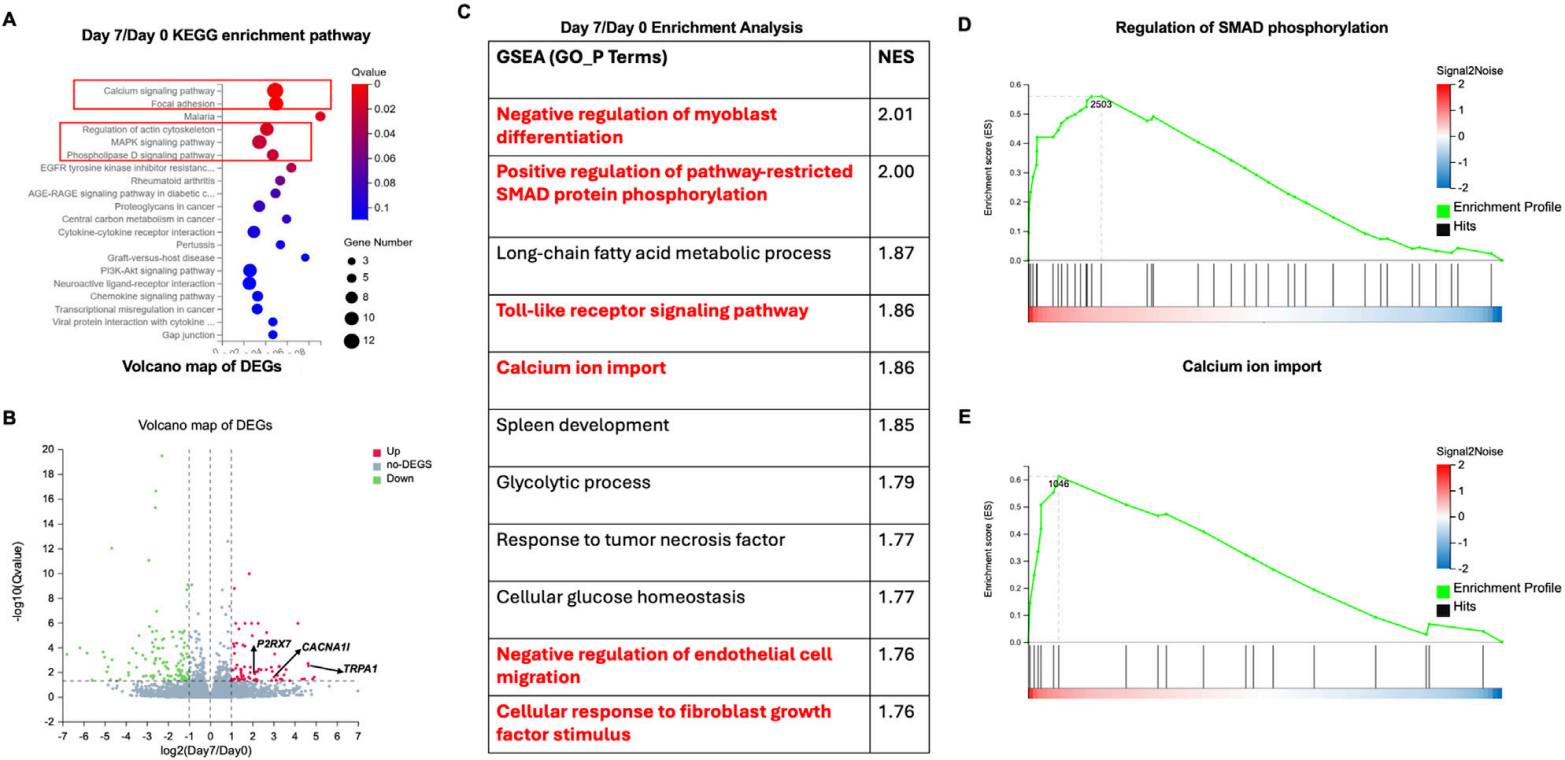
Pathway-level analyses reveal calcium signaling as the most enriched KEGG pathway in Day 7 iMSCs. **(A)** Top KEGG pathways enriched in Day 7 vs. 0 (ranked by enrichment ratio, q ≤ 0.1); red boxes highlight selected calcium signaling pathway and other top KEGG enriched pathways. **(B)** Volcano plot of Day 7 vs. Day 0 DEGs (Log2FC ≥ 1, q ≤ 0.05); selected calcium channel genes are highlighted. **(C)** GSEA enrichment plot for top biological terms between Day 7 vs. Day 0 iMSCs. Selected GSEA enrichment plot for **(D)** positive regulation of pathway-restricted SMAD phosphorylation. **(E)** Calcium ion import. Full KEGG and GSEA enrichment data are provided in Supplementary Tables S5, S6. n = 4 biological replicates per group. Cross-species expression of calcium channel genes in mouse postnatal intestinal mesenchyme (GSE184158) is provided in Supplementary Figures S3-S5.

Among the transcriptional comparisons, the Day 7 iMSCs vs. Day 0 dataset was selected for deeper pathway analyses, given the unique intermediate biological processes associated with this transition period. KEGG profiling revealed that overrepresented terms on Day 7 relative to Day 0 (q-value <0.1) were primarily pathways linked with calcium signaling, focal adhesion, regulation of actin cytoskeleton, MAPK signaling, and phospholipase D signaling (Figure 5A). Calcium channel signaling ranked among the most significantly enriched pathways in Day 7 iMSCs (Figure 5A; Supplementary Table S5), characterized by the upregulation of calcium channel genes (Figure 5B). Corroborating the KEGG findings, the GSEA analyses identified enriched biological processes in Day 7 iMSCs, such as calcium ion import, prostaglandin biosynthetic process, positive regulation of SMAD phosphorylation, positive regulation of angiogenesis, and regulation of chemokine and cytokine production (Figures 5C–E; Supplementary Table S6). Together, these integrated analyses highlight calcium channel-associated genes (*TRPA1*, *CACNA1I*, and *P2RX7*) as candidate signatures among multiple enriched signaling pathways in Day 7 iMSCs compared with neonatal Day 0 iMSCs.

To provide an independent, cross-species context for this calcium channel enrichment in early postnatal intestinal development, we interrogated a publicly available mouse postnatal intestinal mesenchyme scRNA-seq dataset (GSE184158) (McCarthy et al., 2023). Age-dependent expression of *Trpa1, Cacna1i,* and *P2rx7* was observed at the postnatal Day 5 (P5) samples, most prominently in subepithelial mesenchymal cells, including intestinal subepithelial myofibroblasts (ISEMF), trophocytes, and lamina propria muscularis mucosae populations (Supplementary Figures S3, S4). Moreover, Spearman correlation analyses revealed a positive correlation between *Trpa1* (a calcium-permeable ion channel) and lineage-differentiation niche factors, including *Bmp4, Bmp2, Pdgfra,* and *Foxl1* in ISEMF cells (mean rho = 0.17–0.30, *Padj* < 0.001; Supplementary Figure S5). These cross-species observations provide independent, correlative support for a potential relationship between calcium channel-associated genes and niche factor expression during early postnatal intestinal development, complementing the calcium channel gene enrichment identified in piglet Day 7 iMSCs.

### 3.5 Calcium channel inhibition in Day 7 iMSCs is associated with decreased *BMP4* and increased *RSPO3 and WNT* expression

**FIGURE 6:**
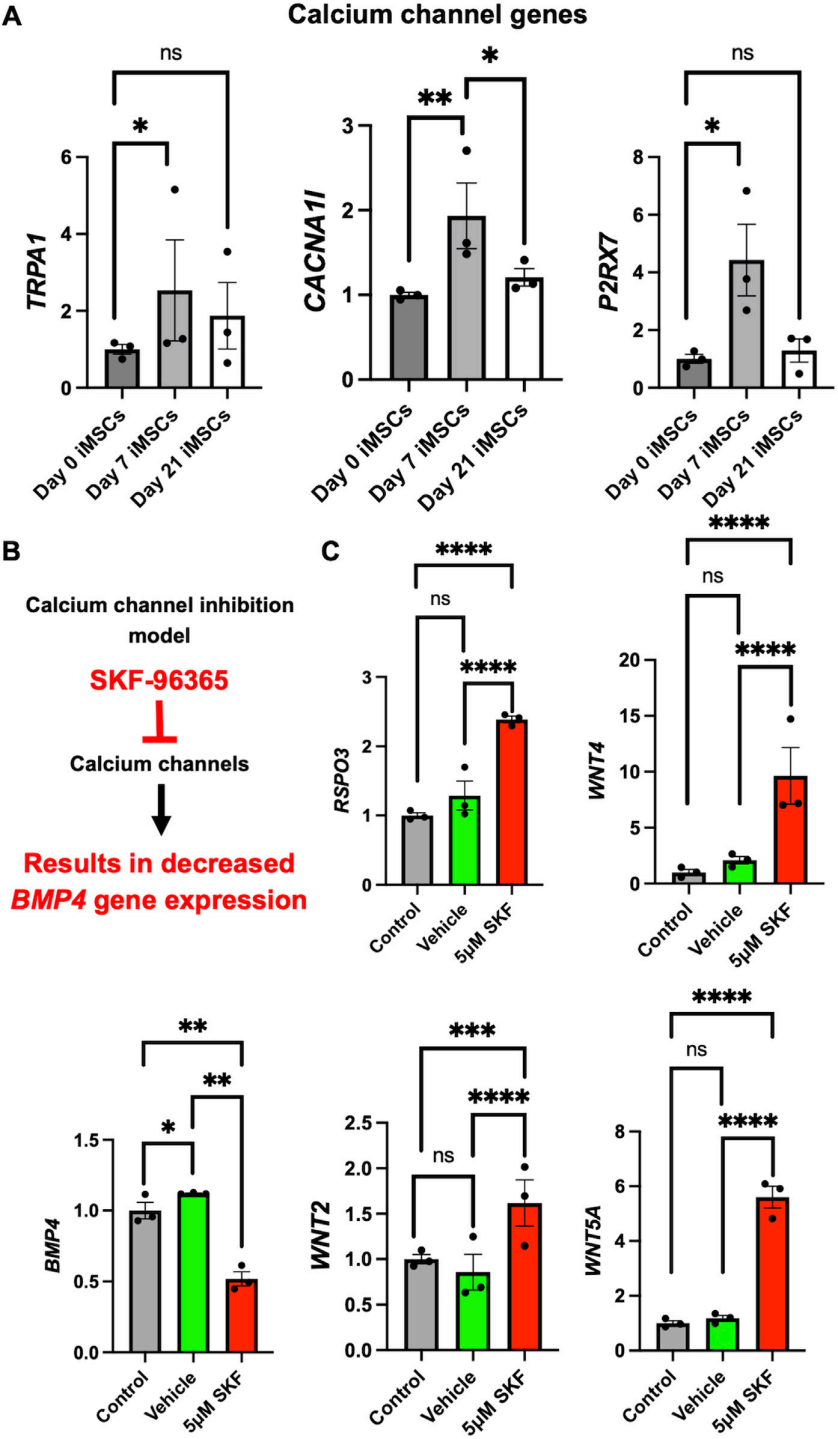
Calcium channel gene expression and effect of short-term calcium channel inhibition in Day 7 iMSCs. **(A)** Relative mRNA expression of calcium channel-associated genes (*TRPA1, CACNA1I, P2RX7)* in Days 0, 7, and 21 iMSCs. Bars show the mean expression ±SEM of n = 3 individual biological replicates per age group. Statistical differences were performed using one-way ANOVA with Dunnett’s multiple comparisons (*p < 0.05, **p < 0.0001) **(B,C)** Relative mRNA expression of niche factor gene in Day 7 iMSCs following 2-h treatment with the non-selective calcium channel blocker SKF-96365 compared with the vehicle-treated controls. Bars show the mean expression ±SEM of n = 3 technical replicates. Statistical comparisons were performed using one-way ANOVA with Sidak’s multiple comparison test (*p < 0.05, **p < 0.002, ***p < 0.0003, ****p < 0.0001). Individual data points are shown for all panels.

Building on the pathway enrichment data, we evaluated the expression of selected calcium channel genes by qPCR and assessed whether pharmacological inhibition of these channels would alter niche factor expression in Day 7 iMSCs (Peiris et al., 2007; Chalmers et al., 2025). qPCR analyses confirmed the significant upregulation of *TRPA1, CACNA1I,* and *P2RX7* in Day 7 iMSCs relative to Day 0 and Day 21 iMSCs, consistent with their enrichment trends observed in the RNA-seq dataset (Figure 6A). A 2 h, short-term pharmacological inhibition with the non-selective calcium channel blocker (SKF-96365) in Day 7 iMSCs was associated with rapid changes in niche factor transcript levels, including a significant decrease in *BMP4*, along with concurrent increase in *RSPO3, WNT2, WNT4,* and *WNT5A* compared with vehicle controls (untreated Day 7 iMSCs) (Peiris et al., 2007; McCarthy et al., 2020; Jacob et al., 2022; Kraiczy et al., 2023) (Figures 6B,C). BMP4 expression, which normally peaks in untreated Day 7 iMSCs (Figure 2A), decreased following calcium channel inhibition, while RSPO3 and WNT ligand expression increased in parallel. This shift resembles the niche factor landscape of neonatal Day 0 iMSCs, suggesting a putative link between calcium channel-responsive pathways and the niche factor balance in preweaning iMSCs. Taken together, these pharmacological, transcriptomic, and cross-species findings collectively suggest a possible link between calcium channel-associated gene regulation and the Day 7 iMSC transcriptional state during early postnatal intestinal development in piglets.

## 4 Discussion

The current study provides the first characterization of iMSCs across the early postnatal period in piglets, revealing that adherent iMSCs exhibit age-associated differences in niche factor gene expression, enteroid growth support capacity, and transcript profile during the preweaning window. Day 7 iMSCs showed an intermediate profile between neonatal Day 0 and later preweaning Day 21 stages. To our knowledge, these temporal transcriptional and functional changes, including the calcium-associated enrichment at Day 7, have not been previously reported in early postnatal iMSCs. Within the scope of the current data, these findings suggest that the piglet iMSC niche shifts progressively from a proliferation-supporting neonatal state toward a differentiation-promoting profile during the preweaning period, consistent with epithelial morphogenesis occurring over the same window (Verdile et al.; Brugger et al., 2020; Meng et al., 2021; Yin et al., 2022)

At the neonatal stage (Day 0), elevated *RSPO3* (Wnt amplifier) expression in iMSCs corresponds with a niche environment that supports epithelial proliferation described in neonatal piglets (Meng et al., 2021). This finding aligns with the robust enteroid expansion and the highest Ki67 proliferation index observed in enteroids cocultured with Day 0 iMSCs (Figures 1C,D), consistent with the dominant, highly proliferative conditions reported in murine postnatal intestine (Jacob et al., 2022; Manieri et al., 2023; Paerregaard et al., 2023). By Day 7, iMSCs exhibited a shift in niche activity. Increased BMP4 and *PDGFRα* expression in Day 7 iMSCs, coupled with the multibudded morphology and intermediate Ki67 proliferation index observed in the cocultured enteroids (Figures 1C,D), is associated with epithelial differentiation activity (Holloway et al., 2021; Jacob et al., 2022; Kraiczy et al., 2023). Recent murine studies have shown that RSPO3 expression gradually becomes restricted to the deep pericryptal axis, whereas BMP-expressing cells, such as PDGFRα^hi-^positive cells in the subvillus region, support epithelial differentiation (McCarthy et al., 2020; Paerregaard et al., 2023; Ayansola et al., 2024). Consistently, the abundant PDGFRα expression observed in the lamina propria of 7-day-old porcine jejunum highlights their increased spatial-temporal support for epithelial cells in the villi axis. Collectively, these findings support the hypothesis that Day 7 represents a transitioning period in iMSC niche cues to shift from predominantly pro-proliferation to pro-differentiation-associated modulations, consistent with previous single-cell epithelial transcriptome dynamics in piglets (McCarthy et al., 2020; Meng et al., 2021; Jacob et al., 2022; Kraiczy et al., 2023). By Day 21, increased *SFRP1* and *GREM1* expression suggests an increasingly complex niche, exhibiting a balance between both pro-proliferating and pro-differentiation demands as reported in a more mature intestine (McCarthy et al., 2020; Kraiczy et al., 2023; Paerregaard et al., 2023). Together, the age-dependent changes in iMSC profiling parallel with epithelial developmental trajectories and thus provide a basis for their interaction studies during postnatal intestinal development in piglets (Yang et al., 2016; Le Bon et al., 2022; Zou et al., 2022; Kraiczy et al., 2023; Wiarda et al., 2023).

Transcriptomic analyses reinforce this temporal progression. The Day 7 iMSCs transcriptome revealed an intermediate profile between the neonatal and late preweaning transcriptional signatures, consistent with a broader developmental shift occurring across the preweaning window. For example, Meng et al. reported that neonatal epithelial cells undergo substantial metabolic and transcriptional changes between Days 0 and 14, including increased glycolysis, mitochondrial activities, and enhanced lipid metabolism (Meng et al., 2021). In the earlier Day 0–7 period, epithelial expansion occurs in the context of abundant maternal support, including colostral antibodies and rapidly establishing maternal-derived microbiota (Peng et al., 2023), so the mucosa can prioritize rapid growth over intrinsic structural and immune specialization. This is further reflected in our DEG analyses, which showed that nearly five times more genes distinguished Day 7 from Day 0 than distinguished Day 21 from Day 7, indicating that the most substantial transcriptional divergence in iMSCs occurs within the first postnatal week. In line with this, our GO and KEGG analyses identified that early postnatal iMSCs are enriched for proliferative and paracrine response programs, whereas transcripts linked to extracellular matrix organization and immune regulation become more prominent between Days 7 and 21. These patterns are consistent with a transcriptional profile shifting from pro-proliferative activity toward providing structural and immune-responsive supports as the intestine develops across the preweaning stages in piglets.

Among the most significantly enriched pathways, calcium channel-associated genes were identified in Day 7 iMSCs, with TRPA1, CACNA1I, and P2RX7 confirmed as significantly upregulated by qPCR (Figure 6A). While the functional significance of calcium signaling in piglet stromal maturation remains to be explored, calcium-regulated processes have been linked to stem cell fate decisions, differentiation, and cytoskeletal remodeling in other models (Ahamad et al., 2021; Chalmers et al., 2025), consistent with the elevated BMP4 expression observed in Day 7 iMSCs. For instance, activation of the extracellular calcium-sensing receptor (CaSR) increases BMP2 secretion in colonic myofibroblast cells (18Co cells) (Peiris et al., 2007), and calcium signaling similarly acts upstream of neural progenitor differentiation (Snoeck, 2020; Chalmers et al., 2025).

We tested this association directly by inhibiting calcium channels in Day 7 iMSCs using SKF-96365, which decreased BMP4 and increased RSPO3, WNT2, WNT4, and WNT5A expression relative to vehicle controls (Figures 6B,C). Although this result supports an association between calcium channel activity and iMSC niche factor expression, the underlying signaling mechanisms remain undefined and will require targeted knockdown of *TRPA1*, *CACNA1I*, or *P2RX7* in future studies.

This association is further supported by our reanalysis of publicly available murine single-cell RNA sequencing data, in which *P2rx7*, *Trpa1*, and *Cacna1i* showed an inflection expression around postnatal Day 5 in intestinal mesenchyme, coinciding with peak crypt morphogenesis and epithelial stem cell niche confinement in mice (McCarthy et al., 2023). While cross-species expression dynamics may differ in magnitude and cell type specificity, this temporal parallel is consistent with the calcium channel-associated gene enrichments observed in Day 7 piglet iMSCs.

Despite the temporal differences in postnatal iMSC functional and transcriptional profiles, several limitations should be considered. First, we relied on a broad stromal marker to characterize adherent iMSCs, without accounting for purity or the spatially and functionally distinct subtypes that have been described in mice and human studies (Koliaraki and Kollias, 2016; Stzepourginski et al., 2017; Greicius et al., 2018; Kim et al., 2020; McCarthy et al., 2020; Holloway et al., 2021; Paerregaard et al., 2023; Ayansola and Jin, 2025) and bulk RNA sequencing may further mask spatially restricted stromal subpopulations during this developmental window. Future studies should incorporate flow cytometry, single-cell transcriptomics, and spatial profiling, such as fluorescence *in situ* hybridization, to resolve this stromal heterogeneity in postnatal piglets. Second, although pharmacological inhibition in the current study suggested an association between calcium channels and niche factor expression, we did not test whether this shift translates into altered functional effects on enteroid growth; an enteroid coculture assay using SKF-96365-treated Day 7 iMSCs would directly address this. Future work should also selectively target candidate calcium channel genes, such as TRPA1, CACNA1I, and P2RX7, to clarify their specific roles in iMSC function during postnatal intestinal development.

In conclusion, this study presents temporal changes in postnatal iMSC profiles using a piglet experimental model. These shifts in iMSC transcriptional signatures correlate with postnatal epithelial development in piglets (Meng et al., 2021) and align with a similar trend in murine mesenchyme (McCarthy et al., 2023), consistent with the view that iMSCs may contribute to epithelial growth regulation during the preweaning period in piglets (Jacob et al., 2022; Brugger and Basler, 2023; Kraiczy et al., 2023; Ayansola et al., 2024). Within this period, Day 7 emerges as a transitional window associated with calcium channel-related transcriptional changes and altered iMSC niche factor expression. Overall, these findings provide a framework for understanding the potential roles of calcium channel-associated processes in iMSCs during postnatal intestinal development.

## Data Availability

The data generated during and/or analyzed for the current study are provided as figures and supplementary data. The raw sequencing data for the RNA-seq experiments presented in this article are uploaded to the NCBI Sequence Read Archive (SRA) under the BioProject accession number: PRJNA1381730.
