# Supplementary material for: Age-dependent functional and transcriptional differences in intestinal mesenchymal stromal cells during early postnatal development in piglets
Source: Front Cell Dev Biol. 2026 Aug 4;14:1847165. doi: 10.3389/fcell.2026.1847165 (PMC13481863; doi:10.3389/fcell.2026.1847165)
Supplement: Supplementary file 1 [file Image1.pdf]

Supplementary Figure S1

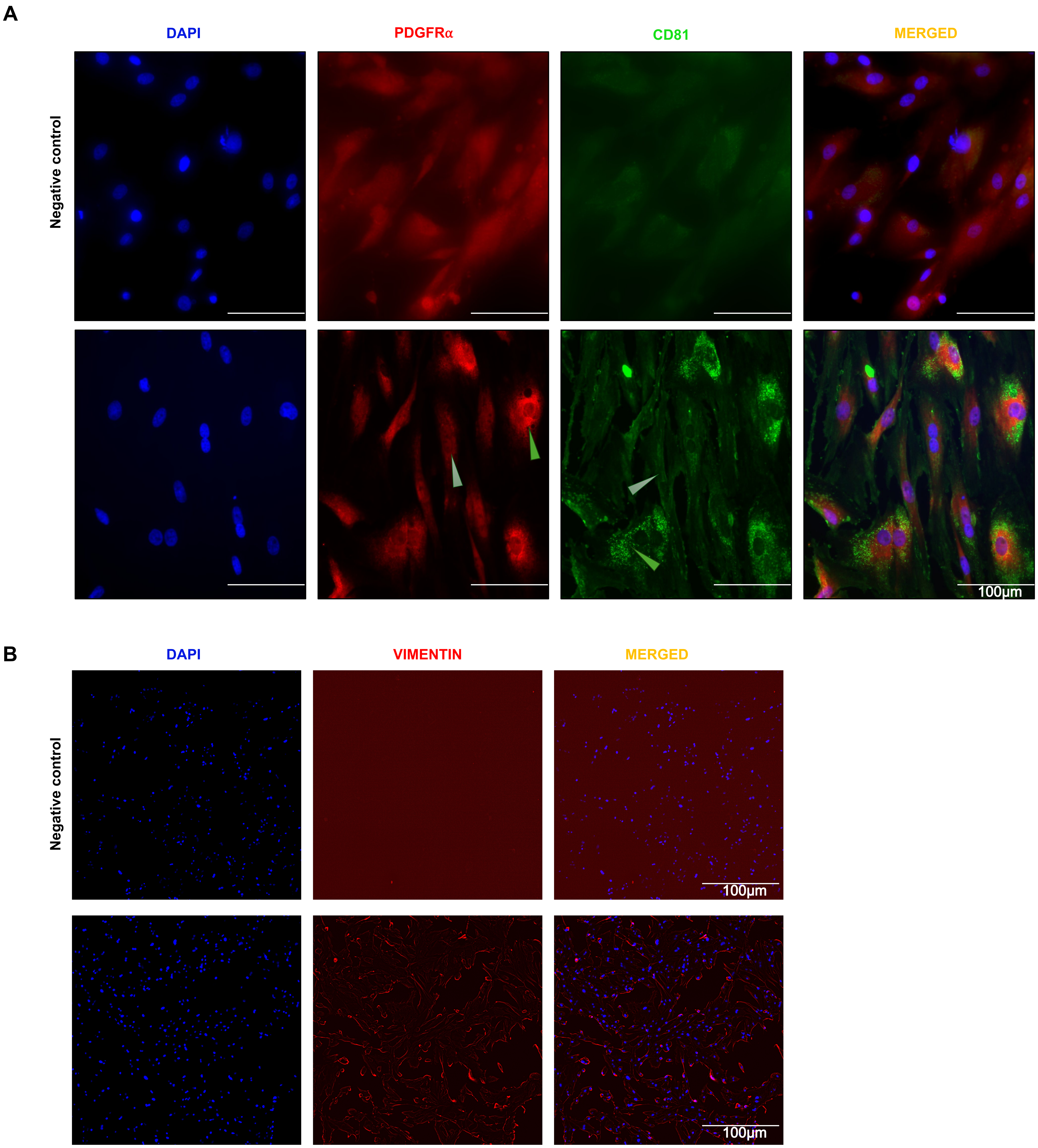

Figure S1: Validation of broad mesenchymal marker expressions in cultured iMSCs. (**A**) Immunofluorescence of cultured piglet jejunal iMSCs at passage 2 (Day 21), stained for PDGFRα (red), CD81 (green), and DAPI (blue), including the negative control panel recently published as a sequential isolation protocol (Hammed and Jin, 2025). (B) Vimentin (red) and DAPI (blue) immunostaining of passage 2 Day 7 iMSCs, confirming mesenchymal cytoskeletal marker expression and characteristic spindle-shaped morphology (Powell et al. , 2011). Scale bar = 100 μm.

Supplementary Figure S2

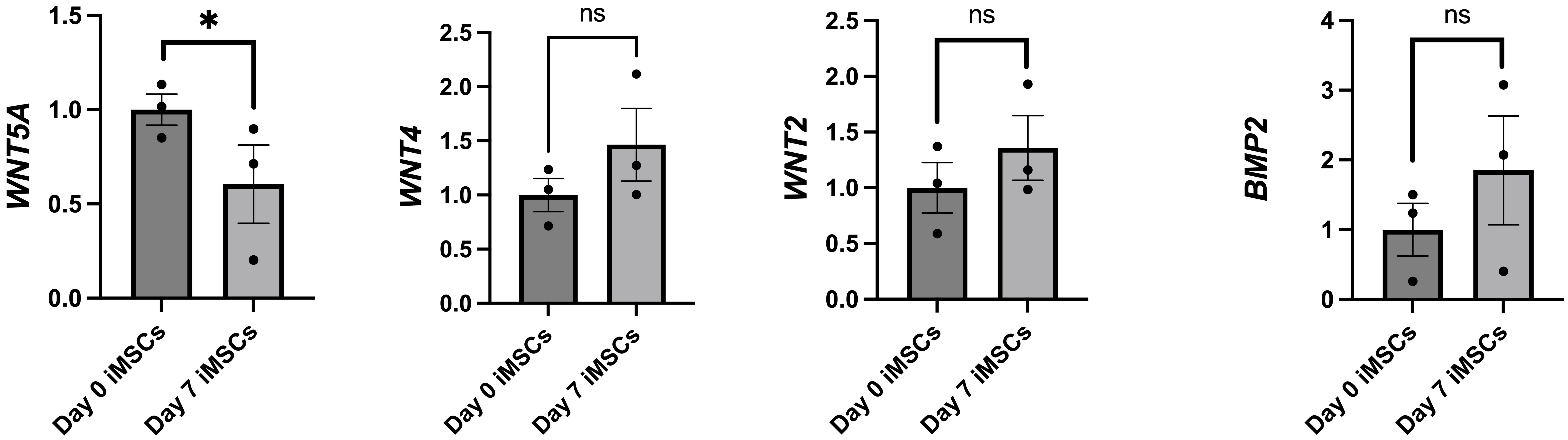

Figure S2: Early postnatal gene expression profile of intestinal mesenchymal stromal cells in the piglet jejunum. Relative mRNA expression (ΔΔCt normalized to reference gene *GAPDH*) of *BMP2*, *WNT4*, *WNT5A*, and *WNT2*. Bars show the mean expression ± SEM of n = 3 independent biological samples. Statistical comparisons were performed using the Mann-Whitney test. *$p<0.01$ and ns represent no statistical differences.

Supplementary Figure S3: Calcium channel expression dynamics in mouse postnatal intestinal stroma cells

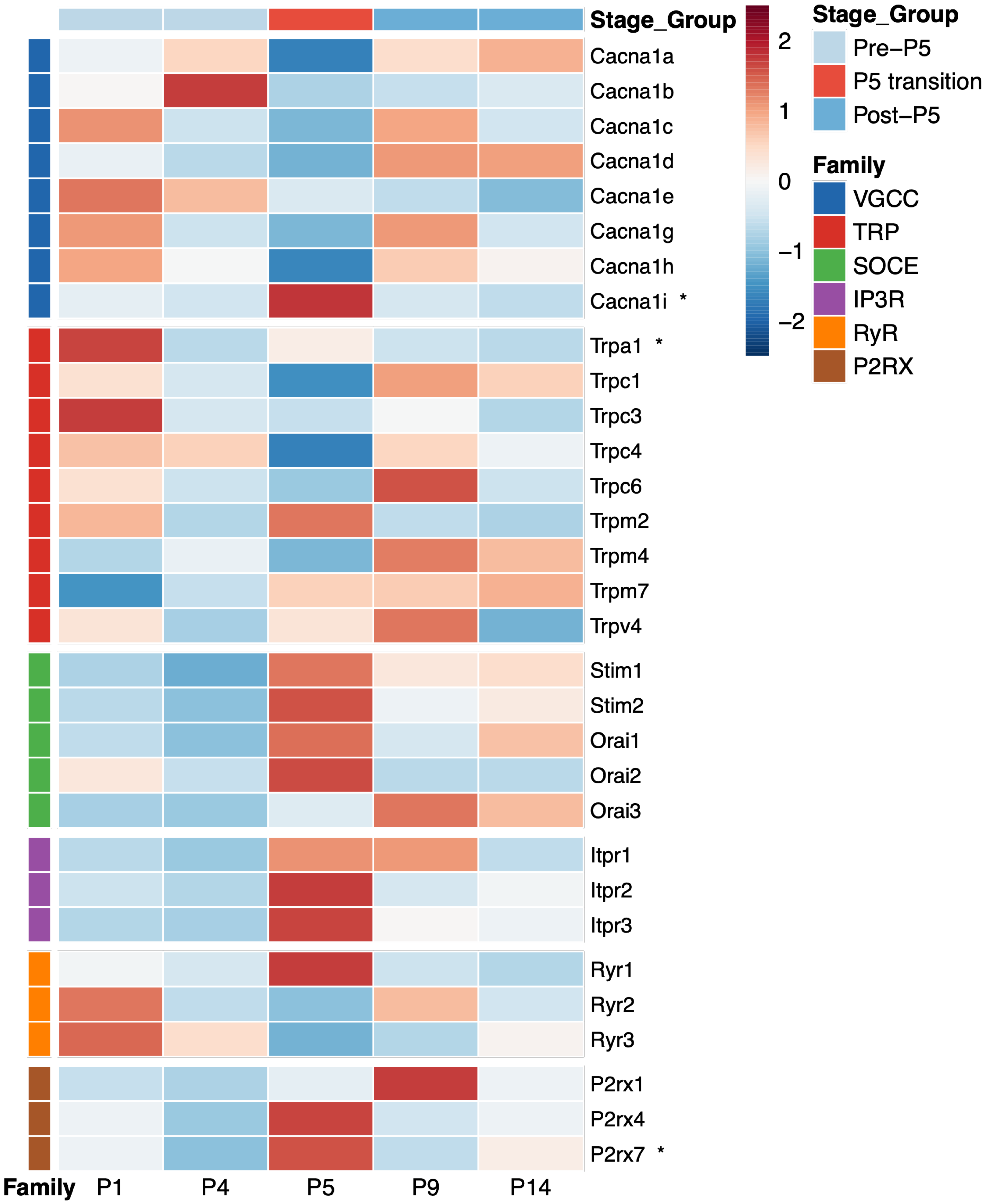

Figure S3: Postnatal developmental dynamics of calcium channel expression across the intestinal stroma in mice.

Heatmap depicting mean normalized expression of 31 calcium channel genes across five postnatal stages (P1, P4, P5, P9, and P14) in pooled intestinal stromal cells from the mouse postnatal intestinal mesenchyme single-cell RNA sequence dataset (GSE184158; McCarthy et al., 2023, Developmental Cell). Genes are grouped by calcium channel family: voltage-gated calcium channels (VGCC; *Cacna1a, Cacna1b, Cacna1c, Cacna1d, Cacna1e, Cacna1g, Cacna1h, Cacna1i*), transient receptor potential channels (TRP; *Trpa1, Trpc1, Trpc3, Trpc4, Trpc6, Trpm2, Trpm4, Trpm7, Trpv4*), store-operated calcium entry channels (SOCE; *Stim1, Stim2, Orai1, Orai2, Orai3*), inositol trisphosphate receptors (IP3R; *Itpr1, Itpr2, Itpr3*), ryanodine receptors (RyR; *Ryr1, Ryr2, Ryr3*), and purinergic receptors (P2RX; *P2rx1, P2rx4, P2rx7*). Genes with a maximum mean expression below 0.02 across all stages were excluded, except *Trpa1* and *Cacna1i*, which were retained due to their selective upregulation in day 7 piglet iMSCs. The postnatal timeline is divided into three developmental windows: pre-P5 (P1 and P4), the P5 transition point, and post-P5 (P9 and P14). **Asterisks (*) denote genes identified as upregulated at Day 7 piglet intestinal mesenchymal stromal cells (iMSCs) in the present study** (*Trpa1, Cacna1i, P2rx7*). Note that *Trpa1* and *Cacna1i* show very low bulk mean expression (max < 0.01) in pooled mouse stromal cells, consistent with their sparse or cell type-restricted expression pattern in mouse intestinal mesenchyme, in contrast to their robust upregulation observed in piglet iMSCs. The color scale represents z-scored mean normalized expression.

Supplementary Figure S4: Cell type-specific calcium channel development trajectories

Mouse postnatal intestinal mesenchyme (GSE184158)

* = upregulated in piglet Day 7 iMSCs (**bold**) | grey = remaining Calcium channel genes | Dash = P5 transition in mouse

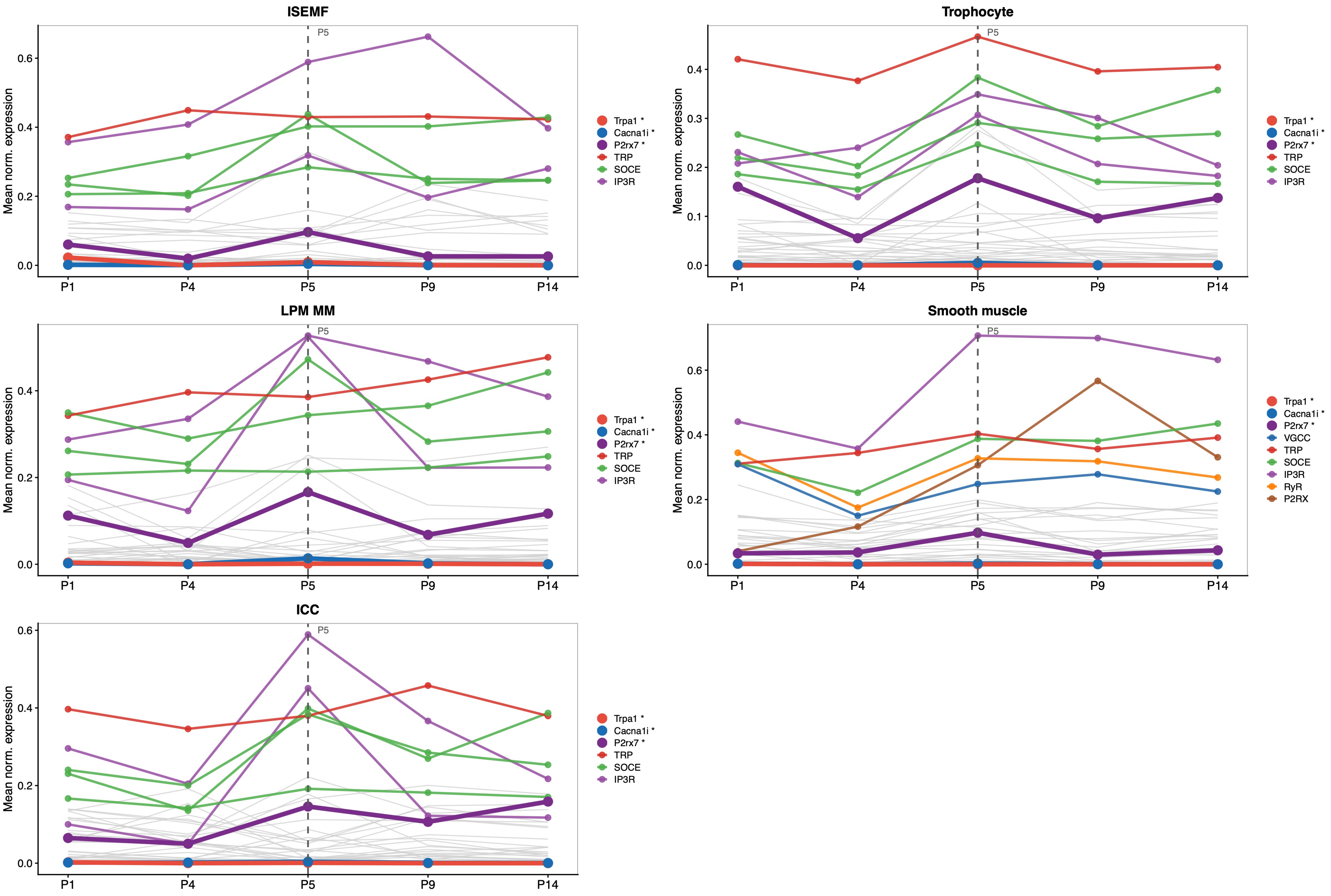

Figure S4: Cell type-specific developmental trajectories of calcium channel expression in mouse postnatal intestinal mesenchyme.

Line plots showing mean normalized expression of all detected calcium channel genes from P1 to P14 across six intestinal mesenchymal cell types: intestinal subepithelial myofibroblasts (ISEMF), trophocytes, lamina-propria-associated myocytes and the muscularis mucosae myocytes (LPM_MM), smooth muscle cells in muscularis externa, interstitial cells of Cajal (ICC), and pericytes. Data are derived from the mouse postnatal intestinal mesenchyme dataset (GSE184158; McCarthy et al., 2023, *Developmental Cell*). The three pig Day 7-upregulated calcium channel genes (*Trpa1*, *Cacna1i*, *P2rx7*; marked with asterisks and shown as bold colored lines) are highlighted against the remaining calcium channel genes shown in grey. Genes are further annotated by channel family as indicated in the legend (VGCC, TRP, SOCE, IP3R, RyR, P2RX). The dashed vertical line marks the P5 developmental transition point, which coincides with the peak of postnatal intestinal crypt morphogenesis. *Note:* The pericyte panel is not shown due to uniformly low or absent expression of the highlighted genes in this cell type.

Supplementary Figure S5: Calcium channel and epithelial niche factor correlation patterns

Mouse postnatal intestinal mesenchyme (GSE184158)

A: Mean Spearman's rho across all stages

Red box= Calcium channel genes (*Trpa1/Cacna1i, P2rx7)* also upregulated in piglet Day 7 iMSCs x niche factors

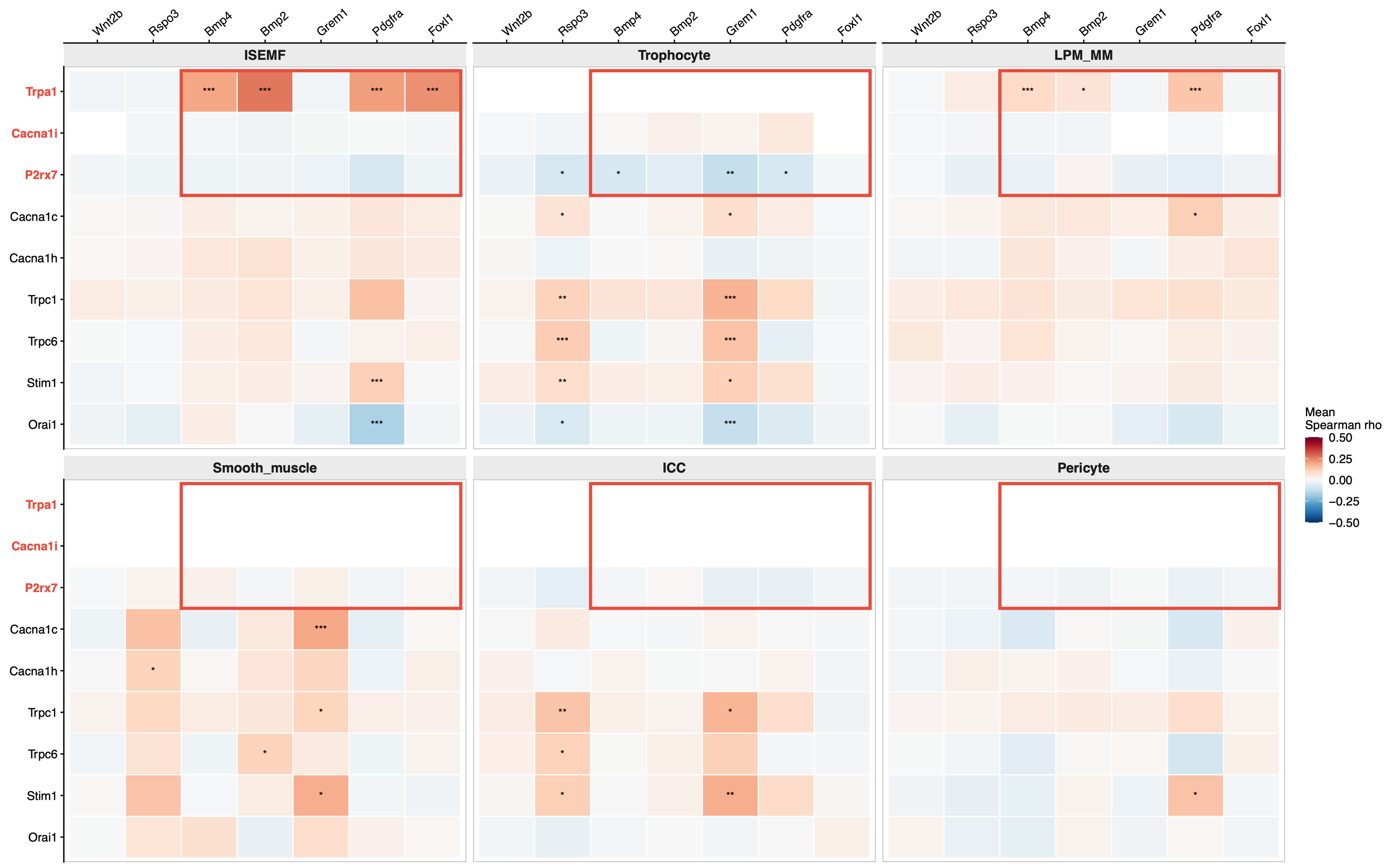

B: P5 stage only (transition point)

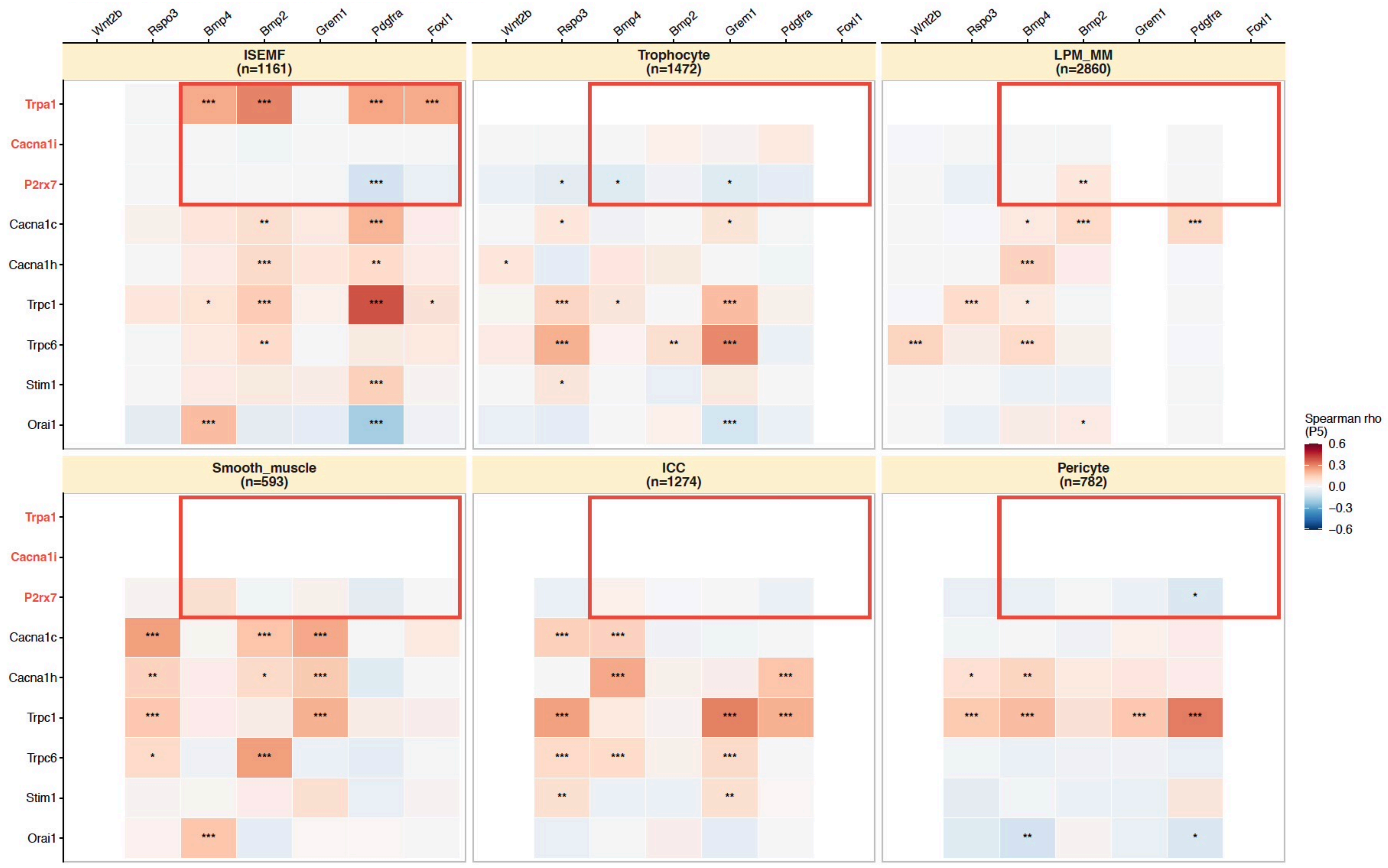

Figure S5: Correlation between calcium channel expression and ISC niche factor co-expression across intestinal mesenchymal cell types.

Heatmaps depicting Spearman correlation coefficients between calcium channel gene expression and intestinal stem cell (ISC) niche factor gene expression (*Wnt2b, Rspo3, Bmp4, Bmp2, Grem1, Pdgfra, Foxl1*) across six intestinal mesenchymal cell types (ISEMF, trophocyte, LPM_MM, smooth muscle, ICC, pericyte) from the mouse postnatal intestinal mesenchyme dataset (GSE184158; McCarthy et al., 2023, *Developmental Cell*). **Panel (A)** shows the mean Spearman rho averaged across all five postnatal stages (P1, P4, P5, P9, P14). Panel **(B)** shows the Spearman rho calculated at the P5 stage alone, representing the critical neonatal transition point at which crypt morphogenesis is maximal. Cell counts per cell type at P5 are indicated. Red boxes highlight the correlations between the three-pig Day 7-upregulated calcium channel genes (*Trpa1, Cacna1i, P2rx7*) and the BMP pathway and stromal identity factors (*Bmp4, Pdgfra, Foxl1*). **Asterisks** denote statistical significance after Benjamini-Hochberg correction (* padj < 0.05, ** padj < 0.01, *** *padj* < 0.001). The color scale represents Spearman rho values; warm colors indicate positive correlations, and cool colors indicate negative correlations.
